# Genome alignment with graph data structures: a comparison

**DOI:** 10.1186/1471-2105-15-99

**Published:** 2014-04-09

**Authors:** Birte Kehr, Kathrin Trappe, Manuel Holtgrewe, Knut Reinert

**Affiliations:** 1 Department of Computer Science, Freie Universität Berlin, Takustr. 9, 14195 Berlin, Germany; 2 Max Planck Institute for Molecular Genetics, Ihnestr. 63-73, 14195 Berlin, Germany

## Abstract

**Background:**

Recent advances in rapid, low-cost sequencing have opened up the opportunity to study complete genome sequences. The computational approach of multiple genome alignment allows investigation of evolutionarily related genomes in an integrated fashion, providing a basis for downstream analyses such as rearrangement studies and phylogenetic inference.

Graphs have proven to be a powerful tool for coping with the complexity of genome-scale sequence alignments. The potential of graphs to intuitively represent all aspects of genome alignments led to the development of graph-based approaches for genome alignment. These approaches construct a graph from a set of local alignments, and derive a genome alignment through identification and removal of graph substructures that indicate errors in the alignment.

**Results:**

We compare the structures of commonly used graphs in terms of their abilities to represent alignment information. We describe how the graphs can be transformed into each other, and identify and classify graph substructures common to one or more graphs. Based on previous approaches, we compile a list of modifications that remove these substructures.

**Conclusion:**

We show that crucial pieces of alignment information, associated with inversions and duplications, are not visible in the structure of all graphs. If we neglect vertex or edge labels, the graphs differ in their information content. Still, many ideas are shared among all graph-based approaches. Based on these findings, we outline a conceptual framework for graph-based genome alignment that can assist in the development of future genome alignment tools.

## Background

Sequence comparison through multiple alignment is an indispensable tool for understanding genomes and their shared histories [1]. Even though the foundation for genomic sequence alignment was already laid in the 1980s [2], the interest is still ongoing [1,3,4], one reason being that it has critical relevance [5] for many bioinformatics analyses. The aim of sequence alignment is to uncover *homologies* by assigning sequence positions to each other, which implies that these positions derived from a common ancestor.

Evolutionary events that change genomic sequences are often classified into small changes and large structural changes [6]. Small changes affect only one or few sequence positions and include substitutions, insertions, and deletions. They do not influence the order of sequence positions, and thus can be captured by *colinear* alignment. Structural changes involve longer genomic segments, thereby affecting the structure and order of genomic sequences. They include *non-colinear* changes like inversions, translocations and duplications in addition to insertions and deletions of longer segments.

While colinear multiple sequence alignment has been studied extensively for a long time [7-16], the problem of non-colinear alignment has been brought into focus only within the last decade [17-22], after more and more whole genomes started to become available. Non-colinear alignments, as opposed to colinear alignments, model all kinds of evolutionary changes and thereby enable correct homology prediction for whole genomes with non-colinear changes. This is comparable to the way global alignments integrate more information than local alignments by assigning all parts of sequences to each other, and the way multiple alignments take information from more than two sequences into account for homology prediction. Over and above, non-colinear multiple global alignments of whole genomes, *genome alignments* for short, integrate as much sequence similarity information as is available.

Together with the prediction of homology, genome alignments provide a *segmentation* of the genomes originating from large structural changes. Depending on the similarity of genomes, segments can be shorter or span several genes and reveal local colinearity. Rearrangement studies [23] explore the order of such segments and infer genomic distances based on the number of breakpoints [24,25] or predict scenarios of evolutionary changes [26-28]. These studies often employ graphs, e. g., breakpoint graphs [29-31], that resemble graph data structures used for genome alignment. Despite this similarity in the approach, genome alignments pursue a slightly different goal than rearrangement studies. The goal is homology prediction instead of reconstruction of evolutionary histories. Genome alignments, which are the focus of this article, integrate more information than rearrangement studies by combining segmentation and sequence similarity.

**Figure 1:**
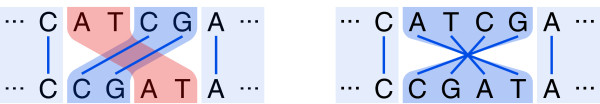
**Alternative alignments of the sequences CATCGA and CCGATA.** The alignment on the left is colinear if the dinucleotides AT (red) are interpreted as insertion or deletion. Alternatively, the AT dinucleotides can be aligned and the CG dinucleotides interpreted as insertion or deletion. Non-colinear aligners that allow for translocations may align the AT dinucleotides in addition to the CG dinucleotides. The alignment on the right shows a non-colinear alternative that interprets the four nucleotides ATCG as inversion (reverse complement). In this example, we expect non-colinear aligners to prefer the inversion (right) over the translocation (left) since it creates fewer segments.

Considering the large search space, genome alignment is an ambitious task and is usually accomplished using heuristic approaches. The first step in genome alignment is commonly the computation of a set of local alignments. It is essential for most methods that the set of local alignments covers all main genomic similarities, whereas additional spurious similarities have a smaller impact. In colinear alignment, such a set usually constitutes a superposition of several alignment possibilities with some local alignments in conflict regarding the colinearity constraint (see Figure 1). The task is then to select the best conflict-free subset according to a given optimization function. In genome alignment, as opposed to colinear alignment, any set of local alignments can be viewed as a valid solution, one that induces a segmentation. However, the induced segmentation can be improved by selecting a subset of local alignments. The subset should contain those local alignments that are most likely to represent homologies when viewed in the context of the whole set of local alignments. The final step is then to find the best segmentation according to the set of local alignments and possibly a subsequent realignment of segments with a colinear alignment method.

For the step of selecting subsets of local alignments and for inducing a segmentation, graphs serve as a convenient tool. The idea is that graphs show substructures indicating errors in the alignment, e. g., specific cycles. Once identified in a graph, we can eliminate these substructures, e. g., by removing local alignments, which is a modification of the genome alignment. Thus, graphs can assist in improving genome alignments. In addition, graphs provide an intuitive representation of similarities and changes between genomes, and so visualize alignment structures. In comparison to tabular alignments, genome alignment graphs are more versatile insofar that it is possible to model colinear and non-colinear changes without the need of choosing a reference genome.

Several graphs have been proposed, each in the context of a specific application such as synteny detection, segmentation, or simply colinear alignment. The earliest graph has been the *alignment graph*, formally defined for colinear multiple alignment by Kececioglu in 1993 [32]. In his definition, the graph contains a vertex for each sequence character and edges for aligned characters. The alignment graph has since been used in various versions, e. g., with additional sequence edges [33] and with genes [34] or segments [15] instead of single characters. In all versions, a colinear alignment can be obtained from the alignment graph by solving the maximum weight trace problem [32], but its structure also allows non-colinear changes to be modeled (see below).

Pevzner et al. introduced *A-Bruijn graphs*[35] as a generalization of de Bruijn graphs [36,37]. The structure of A-Bruijn graphs revisits an idea briefly mentioned by Kececioglu [32], the idea of merging aligned vertices. Consequently, A-Bruijn graphs have one vertex for sets of aligned positions, and edges represent sequence adjacencies. For the purpose of genome alignment with A-Bruijn graphs, the maximum subgraph with large girth (MSLG) problem [19] and the sequence modification problem (SMP) [38] were proposed, both targeting types of short cycles in A-Bruijn graphs in order to eliminate local alignments that hide local colinearity.

In the context of a pipeline for genome alignment that consists of the programs Enredo and Pecan [39], another graph has been published, the *Enredo graph*. The program Enredo applies Enredo graphs to partition genomes into segments. Subsequently, the program Pecan provides nucleotide-level colinear alignments of segments. Enredo graphs have two vertices per set of aligned segments, a head and a tail vertex, resembling breakpoint graphs from rearrangement studies. The Enredo method iteratively eliminates various substructures from the Enredo graph before deriving a final genome segmentation.

A recent and slightly dissimilar graph is the *cactus graph*[22,40]. Cactus graphs have vertices for adjacencies and edges for genome segments. Their structure has two valuable properties. The cactus property subdivides the graph (and genomes) into independent units by ensuring that any edge is part of at most one simple cycle [41]. These units assist in computing genome alignments with the cactus alignment filter (CAF) algorithm [22]. The second property is the existence of an Eulerian circuit. This circuit traverses all genome segments exactly once, even duplicated segments, conveniently providing a consensus genome.

**Figure 2:**
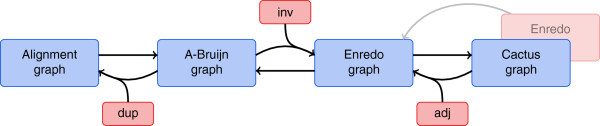
**Overview of transformations among four graph representations for genome alignments.** Some transformations require information from labels (red boxes), which is not present in the graph structures (see text for details). The Cactus method keeps an Enredo-like graph in addition to the cactus graph.

In this paper, we compare the mentioned graph-based genome alignment approaches with an emphasis on the structures of the underlying graphs. Our aim is to clarify similarities of the approaches and the underlying graphs but also to work out differences and highlight limitations. We realize our comparison using the same terminology for all graphs and by describing transformations among the graphs (see Figure 2). We assess the graphs in terms of their capabilities to display alignment information in their structure alone. For all graphs, substructures and modifications constitute key aspects of corresponding genome alignment approaches. We carefully examine substructures as well as modifications independently from the particular graphs they were first described for. Founded on our comparison, we derive a generic framework for graph-based genome alignment. The framework gives an overview of the general graph-based approach to genome alignment and, hence, may assist in the development of future genome alignment tools.

## Results

### Terminology

The biological term *homologous* denotes two or more genomic positions that derived from a single position in an ancestral genome, or two or more segments that derived from a single segment in an ancestral genome. An alignment of genomes is an assignment of positions from the aligned genomes. Usually, the goal is to align only homologous positions to each other, but since the ancestral genome is unknown, an alignment can only be a prediction of homology.

In the following, we formally define a genomic position and give a very general definition of an alignment. Next, we define a genomic segment and constrain the alignment definition to colinearity. Since colinearity is often too strict for predicting homology in whole genomes, genome aligners use so-called blocks, which are colinear alignments of genomic segments. Blocks can be arbitrarily combined to non-colinear genome alignments. We give a general definition of blocks as the basic entities that underlie graph-based genome aligners. Finally, we define the terms adjacency and breakpoint.

Let  be a set of genomes. Each *genome*g∈G is a sequence of characters from the DNA alphabet *Σ*={*A*,*C*,*G*,*T*}. We define the *position* of the (*i*+1)^*s* *t*^ character in genome *g* as *p*=(*g*,*i*) with 0≤*i*<|*g*|, where |*g*| denotes the *length* of *g*. To compare two positions with the operators < and >, we assume an arbitrary strict total ordering of the genomes (such that for any pair of genomes $g_{1},g_{2}\in G$ either *g*_1_<*g*_2_ or *g*_2_<*g*_1_). Then, *p*_1_<*p*_2_ where *p*_1_=(*g*_1_,*i*_1_) and *p*_2_=(*g*_2_,*i*_2_), if *g*_1_<*g*_2_ or if *g*_1_=*g*_2_ and *i*_1_<*i*_2_. Let $P_{G}=\{p|p=(g,i),g\in G,0\leq i<|g|\}$ be the set of all positions of the genomes . An *alignment component* (column) *A* is a subset of $P_{G}$. For example, all pairs of positions connected by a line in Figure 1 form alignment components. Without any demand for optimality, an *alignment* is simply a set of alignment components.

**Figure 3:**
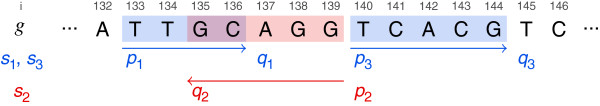
**Three segments of a genome*g*.** Segments *s*_1_=(*p*_1_,*q*_1_) and *s*_3_=(*p*_3_,*q*_3_) are in the forward orientation and their sequences read TTGC and TCACG, respectively. Segment *s*_2_=(*p*_2_,*q*_2_) is in the reverse complemented orientation and reads CCTGC. *s*_1_ and *s*_3_ are non-overlapping but not adjacent, *s*_2_ and *s*_3_ are non-overlapping and adjacent at position *a*=(*g*,140).

An ordered pair of two positions *p*=(*g*,*i*) and *q*=(*g*,*j*) from the same genome *g* defines a *segments*=(*p*,*q*) of length |*i*−*j*|, where min{*p*,*q*} is the smallest position and max{*p*,*q*} the position directly following the largest position in the segment. If *p*<*q*, the segment is in the *forward orientation*, and if *p*>*q*, the segment is in the *reverse complemented* orientation (see Figure 3). As an alternative to an ordered pair (*p*,*q*), a segment could equivalently be represented by a start position, a length, and an additional orientation bit. Two segments *s*_1_=(*p*_1_,*q*_1_) and *s*_2_=(*p*_2_,*q*_2_), where without loss of generality min{*p*_1_,*q*_1_}≤ min{*p*_2_,*q*_2_}, are *non-overlapping* if max{*p*_1_,*q*_1_}≤ min{*p*_2_,*q*_2_}. If max{*p*_1_,*q*_1_}= min{*p*_2_,*q*_2_}, *s*_1_ and *s*_2_ are *adjacent* and define the *adjacency* at position *a*= max{*p*_1_,*q*_1_} (see Figure 3). Two segments *fully overlap* if both min{*p*_1_,*q*_1_}= min{*p*_2_,*q*_2_} and max{*p*_1_,*q*_1_}= max{*p*_2_,*q*_2_}.

An alignment  of a set of segments *S* is *colinear* if each alignment component contains at most one position from each segment *s*∈*S* and if it is possible to impose a strict total ordering ≺ on the alignment components A∈A (such that for any pair of distinct alignment components $A_{1},A_{2}\in A$ either *A*_1_≺*A*_2_ or *A*_2_≺*A*_1_) as follows: The relation *A*_1_≺*A*_2_ holds if for any two positions *p*_1_∈*A*_1_ and *p*_2_∈*A*_2_ from the same segment *s*∈*S*, *p*_1_<*p*_2_ if *s* is in the forward orientation and *p*_2_<*p*_1_ if *s* is in the reverse complemented orientation. If an alignment violates the conditions for colinearity, it is *non-colinear* (see Figure 1). To put it simply, inversions, duplications, and translocations of parts of the aligned sequences are non-colinear operations that violate colinearity.

Non-colinear operations divide an alignment into units that are colinear in themselves but not with respect to each other. We call these units *blocks* and define a block as a colinear alignment of a set of segments. Note that a block may contain multiple segments of the same genome if duplications are present. We refer to the number of segments in a block as the *size* of a block (not to be confused with the *length* of segments). In Figure 1, areas shaded in blue and red indicate blocks. For example in the left alignment, the two dinucleotides CG form a block and the two dinucleotides AT form another block. In the right alignment of Figure 1, the segment ATCG and its reverse complement in the second sequence form a block.

A block always has two equivalent representations. In the first block representation, some segments are in the forward orientation and some may be in the reverse complemented orientation. In the second block representation all segments are in the reverse complemented orientation that are in the forward orientation in the first block representation and all segments are in the forward orientation that are in the reverse complemented orientation in the first block representation. The essential information about possible inversions is the orientation of segments with respect to each other and not the orientation of the block representation. Once we choose one of the two representations, we implicitly assign a tail and a head to a block *b*. The *head* is the set of positions {*p*} of all segments *s*∈*b* with *s*=(*p*,*q*), and the *tail* is the set of positions {*q*}. We refer to the two sets as the *ends* of *b* in cases where the orientation of a block is not given.

A set of blocks constitutes a genome alignment and is input for building a genome alignment graph. To simplify the exposition of the graphs below, we define $B_{G}$ as a set of blocks that is a tiling of the genomes : All pairs of blocks $b_{1},b_{2}\in B_{G}$ have to be non-overlapping; for unaligned segments between blocks and unaligned segments at the ends of the genomes, $B_{G}$ includes blocks of size 1.

Two blocks $b_{1},b_{2}\in B_{G}$ are *adjacent* if there are two segments *s*_1_∈*b*_1_ and *s*_2_∈*b*_2_ that are adjacent. Since all blocks have two ends, there may be up to four different adjacencies between two blocks: the head of *b*_1_ can be adjacent to the head of *b*_2_ or to the tail of *b*_2_, or the tail of *b*_1_ can be adjacent to the head of *b*_2_ or to the tail of *b*_2_. Each of the four adjacencies is defined by a set of adjacency positions between segments from the two blocks, e. g., if the tail of *b*_1_ is adjacent to the head of *b*_2_, the adjacency is defined by the set of positions {*q*_1_} of segments *s*_1_∈*b*_1_ with *s*_1_=(*p*_1_,*q*_1_) for which there is a segment *s*_2_∈*b*_2_ with *s*_2_=(*p*_2_,*q*_2_) where *p*_1_=*q*_2_. Since a block can contain more than one segment from the same genome, a block can be adjacent to itself. In the literature, adjacencies of blocks are sometimes defined as segments between two blocks [39]. Given that the set of blocks $B_{G}$ is a tiling of the genomes, we can refer to an adjacency as a set of single positions.

An adjacency of two blocks $b_{1},b_{2}\in B_{G}$ is called a *breakpoint* if *b*_1_ and *b*_2_ are adjacent in only a subset of the segments. Then, the set of positions that define the adjacency is smaller than the size of the block. More formally, let *s*_1_∈*b*_1_ and *s*_2_∈*b*_2_ be two adjacent segments with *s*_1_=(*p*_1_,*q*_1_) and *s*_2_=(*p*_2_,*q*_2_). Without loss of generality, let *q*_1_=*p*_2_. Then, *b*_1_ and *b*_2_ define a breakpoint if there is a segment $s_{1}^{\prime }\in b_{1}$ with $s_{1}^{\prime }=(p_{1}^{\prime },q_{1}^{\prime })$ for which no segment $s_{2}^{\prime }\in b_{2}$ with $s_{2}^{\prime }=(p_{2}^{\prime },q_{2}^{\prime })$ exists where $q_{1}^{\prime }=p_{2}^{\prime }$.

Most commonly, genome alignment programs use pairwise local alignment methods to generate blocks. Pairwise local alignments are blocks of size two. These blocks can be combined with each other to form blocks of a larger size (multiple local alignments) if a segment from one block fully overlaps with a segment from another block. We briefly address this preprocessing of blocks in the Discussion and conclusions section, and assume that a set $B_{G}$ is given for constructing the graphs.

In the literature, blocks are often referred to as synteny blocks or locally colinear blocks. The definitions of blocks differ, usually depending on the specific type of local alignment method being used for generating blocks. For example, blocks can be defined as gapped or ungapped colinear alignments with or without mismatches, or simply as single alignment components. The graph representations are independent from the precise assignment of positions to alignment components within blocks. Only the set of segments including their relative orientation within the block is relevant. For this reason, the different block definitions can be used interchangeably except for preprocessing the set of blocks to obtain $B_{G}$ (see Discussion and conclusions section).

Within the graphs described in the following sections, blocks and adjacencies are represented by vertices or edges or a combination of both. For each graph, every genome is a (not necessarily simple) path through the graph. We use the term *to thread* for following the path of a genome through the graph [17,35].

### Graphs for genome alignment

**Figure 4:**
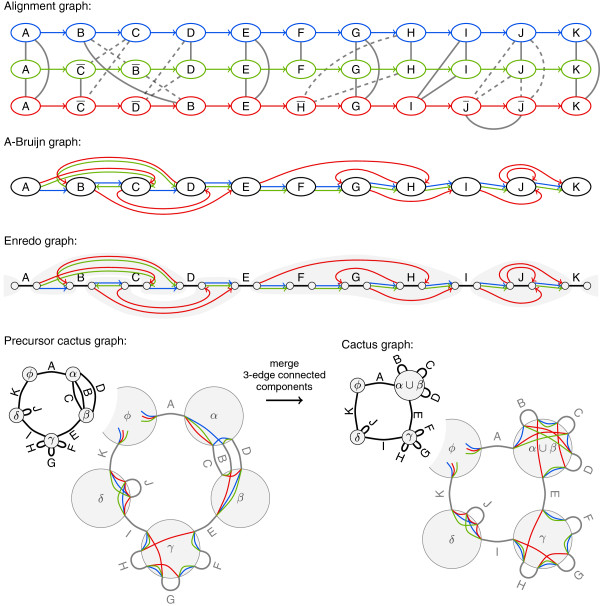
**An alignment of three genomes with eleven blocks in all four graph representations.** The example covers multiple non-colinear events: Blocks A, E, I, K are conserved in all three genomes without large structural changes. Blocks B, C, and D, as well as G, and H appear in different orders and orientations. Block F is missing in the red genome and J occurs twice. Colors denote the three genomes. In the alignment graph, dashed edges indicate the alignment of a segment with its reverse complement. We consider the information provided by line styles not to be part of the graph structures. In the Enredo graph, components connected by adjacency edges are shaded in gray. For the cactus graph, the figure additionally shows a precursor. Furthermore, enlarged vertices for the precursor and the final cactus graph show adjacencies in vertices.

We limit our comparison to alignment graphs, A-Bruijn graphs, Enredo graphs, and cactus graphs. The original publications of these graphs use varying terminology. We describe all four graphs using the same terminology, namely the above defined terms segment, block and adjacency. Figure 4 displays an example alignment with eleven blocks as alignment graph, A-Bruijn graph, Enredo graph, and cactus graph.

#### 

The input for building a graph is a set of non-overlapping blocks $B_{G}$ defined on a given set of genomes . We assume the blocks to be a tiling of . For all four graphs, we define the *graph structuresG*=(*V*,*E*) as ordered pairs of vertices *V* and edges *E*. In addition, we define *graph modelsM*_*G*_=(*G*,*ℓ*), which are ordered pairs of the respective graph structure *G* and a *labeling functionℓ*. Most original publications remain vague about labels on vertices and edges of the graph structures. We define *ℓ* such that the set of blocks $B_{G}$ can be recovered from *M*_*G*_.

Along with the definitions of *G* and *M*_*G*_ for each of the four graphs, we describe how it is possible to transform the different graph structures into each other (e. g., an alignment graph structure into an A-Bruijn graph structure). A *transformation* is an operation that has as input one graph structure *G* and outputs another graph structure *G*^′^, where both *G* and *G*^′^ represent the same genome alignment. If it is possible to obtain a graph structure *G*^′^ from another graph structure *G* without the help of $B_{G}$ and without a labeling function, then *G* provides at least as much alignment information as *G*^′^. Moreover, if a graph structure *G* provides less information about the alignment than another graph structure *G*^′^, a transformation from *G* to *G*^′^ is ambiguous, thus, impossible.

We examine the transformations that are depicted as arrows in Figure 2. Straight arrows indicate a possible transformation; the other arrows indicate that a transformation among the structures is impossible, which we prove below by providing examples for ambiguity. Nevertheless, we describe all transformations depicted as arrows in Figure 2, using additional information from graph labels if necessary to resolve ambiguity. We define the sparse labeling functions *ℓ*^*d* *u* *p*^, *ℓ*^*i* *n* *v*^, or *ℓ*^*a* *d* *j*^ for this purpose. The sparse labeling functions provide sufficient information for the transformation but less information than *ℓ* in the graph models. Note that a transformation among graph models is trivial given that $B_{G}$ can be recovered from the model *M*_*G*_ of any of the four graphs. The need for labels to resolve ambiguity proves that the graph structures *G* differ in their information content.

##### Alignment graphs

In the following section, let *G*=(*V*,*E*) be an alignment graph structure and *M*_*G*_=(*G*,*ℓ*) be an alignment graph model. We define *ℓ* as a labeling function of the vertices *V* of *G*. The set of edges *E*=*E*_*A*_∪*E*_*B*_ decomposes into a set of directed adjacency edges *E*_*A*_ and a set of undirected block edges *E*_*B*_. With both directed and undirected edges, *G* is a *mixed graph*.

The vertices *V* of *G* represent segments of the genomes. There is a vertex in *V* for every segment in the set of all segments ($S_{B}=\underset{b\in B_{G}}{⋃}b$) from all blocks $B_{G}$. The function *ℓ*:*V*→*S*_*B*_ labels each vertex *v*∈*V* with the corresponding segment *s*∈*S*_*B*_ such that *ℓ*(*v*)=*s*.

Directed adjacency edges *E*_*A*_ (colored edges in Figure 4) represent adjacencies of segments. Given any pair of vertices *v*_1_,*v*_2_∈*V* and their labels *ℓ*(*v*_1_)=(*p*_1_,*q*_1_) and *ℓ*(*v*_2_)=(*p*_2_,*q*_2_), there is a directed edge *e*∈*E*_*A*_ from *v*_1_ to *v*_2_ if max{*p*_1_,*q*_1_}= min{*p*_2_,*q*_2_}, i. e., the segment *ℓ*(*v*_2_) is adjacent to the segment *ℓ*(*v*_1_) in . Adjacency edges thread the genomes through the alignment graph.

Finally, undirected block edges *E*_*B*_ (gray edges in Figure 4) connect vertices labeled with segments from the same block $b\in B_{G}$. For each vertex *v*_1_∈*V* with *ℓ*(*v*_1_)∈*b*, there are undirected edges *E*_*B*_ between *v*_1_ and any other vertex *v*_2_∈*V* with *ℓ*(*v*_2_)∈*b*. As a consequence, each block $b\in B_{G}$ forms a *block edge connected componentC*⊆*V* in the alignment graph.

The formation of connected components is important for recovering $B_{G}$ from *M*_*G*_. Let  be the set of block edge connected components of *G*. It holds $V=\underset{C\in C}{⋃}C$, and *C*_1_∩*C*_2_=*∅* for any $C_{1},C_{2}\in C$. Thus, we may recover $B_{G}$ by forming a block *b*={*ℓ*(*v*)∣*v*∈*C*} for every component C∈C.

Our definition of the alignment graph structure *G* models non-colinear changes among the input genomes, in particular translocations and duplications. Translocations appear in *G* as mixed cycles. A *mixed cycle* is a cycle in a mixed graph formed by both directed and undirected edges. Duplications appear as block edges within the set of vertices of one genome. Because of these edges our alignment graph is not n-partite as in its original definition [32].

**Figure 5:**
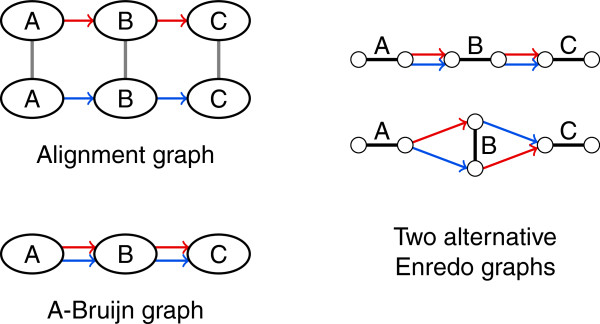
**The structure of alignment graphs and A-Bruijn graphs does not display inversions.** Let $\bar{B}$ denote the reverse complemented block representation of a block *B*. The alignment graph structure and A-Bruijn graph structure for the two sequences ABC and A$\bar{B}$C is the same as for the two sequences ABC and ABC. The orientation of blocks remains unclear in the graph structures. However, there are two Enredo graph structures for the alternative orientations of block B in one sequence. Thus, the transformation from the alignment graph structure or A-Bruijn graph structure to an Enredo graph structure is ambiguous with two alternatives.

Inversions are not visible in the alignment graph structure *G*; the orientation of segments remains unclear (see also Figure 5). We define the sparse labeling function *ℓ*^*i* *n* *v*^:*V*→{+,−} as

$$\ell^{\text{inv}}(v)=\left\{\begin{array}{cc} + & \text{if}\;\;p<q\\ - & \text{if}\;\;p>q, \end{array}\right)$$

where *ℓ*(*v*)=(*p*,*q*). The function *ℓ*^*i* *n* *v*^ assigns bits to the vertices that indicate the orientation of the represented segments. As an alternative to vertex labels, it is possible to label block edges with bits that indicate equal or opposite orientation of the segments in the endpoints (visualized as dashed and solid lines in Figure 4 or red and black edges in [42]).

##### A-Bruijn graphs

Let now *G*=(*V*,*E*) be an A-Bruijn graph structure and *M*_*G*_=(*G*,*ℓ*) be an A-Bruijn graph model. A-Bruijn graphs have only one type of edge *E*. We define *ℓ* as a labeling function of the vertices *V*. In contrast, the functions *ℓ*^*i* *n* *v*^ and *ℓ*^*d* *u* *p*^ described below provide labels for the edges *E*.

The vertices *V* of *G* represent blocks. For every block in $B_{G}$, there is a vertex in *V*. There is only one vertex per block regardless of the block’s size and of duplications. The function $\ell :V\to B_{G}$ labels each vertex *v*∈*V* with the corresponding block $b\in B_{G}$ such that *ℓ*(*v*)=*b*. With this labeling, recovering $B_{G}$ from *M*_*G*_ is straightforward.

The edges *E* of *G* represent adjacencies just like adjacency edges in alignment graphs. Given any pair of vertices *v*_1_,*v*_2_∈*V* and their labels *b*_1_=*ℓ*(*v*_1_) and *b*_2_=*ℓ*(*v*_2_), there is a directed edge *e*∈*E* from *v*_1_ to *v*_2_ for every two adjacent segments *s*_1_=(*p*_1_,*q*_1_) and *s*_2_=(*p*_2_,*q*_2_) with max{*p*_1_,*q*_1_}= min{*p*_2_,*q*_2_} where *s*_1_∈*b*_1_ and *s*_2_∈*b*_2_. If multiple adjacent pairs of segments exist in *b*_1_ and *b*_2_, *E* contains multiple edges from *v*_1_ to *v*_2_. Thus, *G* is a multi-graph. In the present paper, we prefer the multi-graph representation with multiple separate edges between two vertices over the multi-graph representation with multiplicity labels on edges.

**Figure 6:**
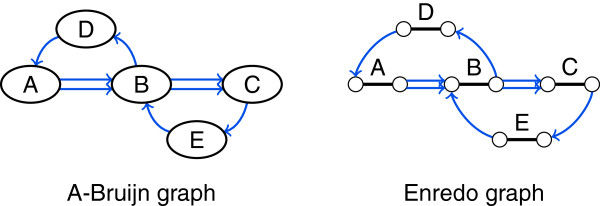
**Duplications may create ambiguity in the structure of A-Bruijn graphs and Enredo graphs.** In this example, the structure of the A-Bruijn graph and the Enredo graph represents both the genomes AB**DA**B**CE**BC and AB**CE**B**DA**BC. Thus, the order of blocks is ambiguous.

Adjacency edges are essential for threading genomes through *G*. However, the path from threading one genome is not necessarily simple. It traverses vertices multiple times if duplications are present (see block J in Figure 4) making the path ambiguous. Thus, threading requires label information that allows incoming and outgoing edges of a vertex to be paired. Such information is not required in the alignment graph structure, where each vertex has at most one incoming and one outgoing edge. Without duplications it is sufficient to color edges of *G* by genome (red, blue, and green in Figure 4) instead of providing the full labels *ℓ*. In the presence of duplications, *G* can be ambiguous even with color labels (see Figure 6 and block J in Figure 4).

To resolve ambiguity of *G* for threading, we define the sparse labeling function $\ell^{\text{dup}}:E\to \mathbb{N}$ as a total ordering on the edges. This function assigns numbers to the edges that describe the order of the adjacencies in the genomes. In Figure 6, for example, the edges could be numbered 1 through 8: for genome ABDABCEBC, one edge from A to B would be labeled with 1, the edge from B to D would be labeled with 2, the edge from D to A would be labeled with 3, and so on; for genome ABCEBDABC, also one edge from A to B would be labeled with 1, but then the edge from B to C would be labeled with 2, and so on. To describe *ℓ*^*d* *u* *p*^ formally, we use adjacency positions of the edges *E* that we determine with the help of *ℓ*. Given a pair of vertices *v*_1_,*v*_2_∈*V*, each edge *e*∈*E* from *v*_1_ to *v*_2_ corresponds to one pair of adjacent segments *s*_1_∈*ℓ*(*v*_1_) and *s*_2_∈*ℓ*(*v*_2_) with *s*_1_=(*p*_1_,*q*_1_) and *s*_2_=(*p*_2_,*q*_2_). The adjacency position of *e* is *a*= max{*p*_1_,*q*_1_}= min{*p*_2_,*q*_2_}. Then, *ℓ*^*d* *u* *p*^ assigns numbers to edges in *E* such that

$$\ell^{\text{dup}}(e_{1})<\ell^{\text{dup}}(e_{2})\;\text{if}\;a_{1}<a_{2},$$

where *a*_1_ is the adjacency position of *e*_1_, and *a*_2_ is the adjacency position of *e*_2_.

Furthermore, inversions create ambiguity in *G* (see Figure 5). Just like the alignment graph structure, *G* provides no information about the orientation of segments represented in a vertex. We define the sparse labeling function *ℓ*^*i* *n* *v*^:*E*→{+,−}×{+,−} for A-Bruijn graph edges *E*. For each pair of adjacent segments *s*_1_=(*p*_1_,*q*_1_) and *s*_2_=(*p*_2_,*q*_2_) from the labels of two vertices *s*_1_∈*ℓ*(*v*_1_) and *s*_2_∈*ℓ*(*v*_2_), we label an edge *e*=(*v*_1_,*v*_2_) with

$$\ell^{\text{inv}}(e)=\left\{\begin{array}{cc} \left(+,+\right) & \text{if}\;\;p_{1}<q_{1}\wedge p_{2}<q_{2},\\ \left(+,-\right) & \text{if}\;\;p_{1}<q_{1}\wedge p_{2}>q_{2},\\ \left(-,+\right) & \text{if}\;\;p_{1}>q_{1}\wedge p_{2}<q_{2},\\ \left(-,-\right) & \text{if}\;\;p_{1}>q_{1}\wedge p_{2}>q_{2}. \end{array}\right)$$

**Figure 7:**
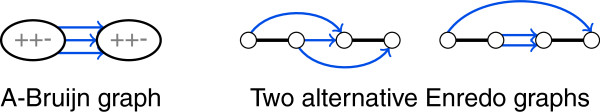
**The labeling of A-Bruijn graph vertices with one orientation bit per segment does not resolve ambiguity.** In this example, both blocks occur three times, twice in the forward orientation and once in the reverse complemented orientation. Combining the orientations of the segments in the two blocks is ambiguous as the two alternative Enredo graph structures prove. In the left Enredo graph structure, the segment in the reverse complemented orientation of one block is combined with a segment in the forward orientation of the other block. In the right Enredo graph structure, the two segments in the reverse complemented orientation occur consecutively.

The first bit in the label *ℓ*^*i* *n* *v*^(*e*) indicates the orientation of the segment in the source vertex of *e*, and the second bit the orientation of the segment in the target vertex. It is not sufficient to solely label vertices of *G* with one orientation bit per segment of the represented block. Figure 7 provides an example where this leads to ambiguity.

Below, we describe transformations between A-Bruijn graphs and alignment graphs. As stated above, the transformation of the graph *models* is trivial, but the example in Figure 6 proves that in some cases it is impossible to transform an A-Bruijn graph *structure* into an alignment graph structure. We describe the transformation with the help of the sparse labeling function *ℓ*^*d* *u* *p*^ to resolve ambiguity.

###### *A-Bruijn graphs from alignment graphs.*

To transform an alignment graph structure $G^{\prime }=(V^{\prime },E_{B}^{\prime }\cup E_{A}^{\prime })$ into an A-Bruijn graph structure *G*=(*V*,*E*), we follow the description of A-Bruijn graphs in [35] and exploit a many-to-one mapping from alignment graph vertices to A-Bruijn graph vertices. The transformation is possible without additional information from a labeling function.

As a first step, compute all block edge connected components  of *G*^′^. As described above, each component represents exactly one block, and each vertex *v*^′^∈*V*^′^ is part of exactly one component. Now, add for every component C∈C a vertex to the set of A-Bruijn graph vertices *V*. We obtain a many-to-one mapping of alignment graph vertices to A-Bruijn graph vertices. We keep the mapping as a label *m*[ *v*^′^]=*v* on each alignment graph vertex *v*^′^∈*V*^′^. The label indicates the A-Bruijn graph vertex *v* that represents the connected component containing *v*^′^.

The remaining task is to transfer adjacency edges from the alignment graph to the A-Bruijn graph. Using the mapping, add an edge *e*=(*u*,*v*) to the set of A-Bruijn graph edges *E* for each edge *e*^′^=(*u*^′^,*v*^′^) from the set of alignment graph adjacency edges $E_{A}^{\prime }$, where *u*=*m*[ *u*^′^] and *v*=*m*[ *v*^′^].

###### *A-Bruijn graphs to alignment graphs.*

We describe the transformation of an A-Bruijn graph structure *G*=(*V*,*E*) into an alignment graph structure $G^{\prime }=(V^{\prime },E_{A}^{\prime }\cup E_{B}^{\prime })$ given the labeling function *ℓ*^*d* *u* *p*^ for the edges of *G* in addition to *G*. In accordance with the transformation from *G*^′^ to *G*, we successively create a one-to-many mapping from A-Bruijn graph vertices to alignment graph vertices during the transformation. The mapping *m* labels each A-Bruijn graph vertex *v*∈*V* with a set of alignment graph vertices from *V*^′^. At the beginning *m*[ *v*]={} for all *v*∈*V*. At the end, a label $m[v]=\{v_{1}^{\prime },\ldots ,v_{\left|b\right|}^{\prime }\}$ indicates the set of alignment graph vertices $\left\{v_{1}^{\prime },\ldots ,v_{\left|b\right|}^{\prime }\right\}$ that form the connected component for a block *b* represented by *v* in the A-Bruijn graph.

We transform the A-Bruijn graph by following each genome separately and assume that the edges are given in increasing order of labels: *ℓ*^*d* *u* *p*^(*e*_1_)<*ℓ*^*d* *u* *p*^(*e*_2_)<⋯<*ℓ*^*d* *u* *p*^(*e*_|*E*|_). Initially, add for each genome a new vertex *u*^′^ to the set of alignment graph vertices *V*^′^. If the source vertex *u* of the A-Bruijn graph edge *e*_1_=(*u*,*v*) is labeled with a non-empty set of vertices $m[\;u]=\{u_{1}^{\prime },\ldots ,u_{k}^{\prime }\}$, add undirected edges between *u*^′^ and all vertices $u_{1}^{\prime },\ldots ,u_{k}^{\prime }$ to the set of alignment graph block edges $E_{B}^{\prime }$. Next, add *u*^′^ to the set of vertices mapped to *u*. Repeat these three steps for the target vertex *v* of *e*_1_: add a vertex *v*^′^, add block edges, and add *v*^′^ to the mapping. In addition, add a directed edge from *u*^′^ to *v*^′^ to the set of alignment graph adjacency edges $E_{A}^{\prime }$.

Iterate over the A-Bruijn graph edges in increasing order of labels and repeatedly add for the target vertex a new vertex, add block edges, add the new vertex to the mapping, and add an adjacency edge from the previous to the new vertex. This way, the genomes are threaded through the A-Bruijn graph and the alignment graph structure *G* is successively built up.

##### Enredo graphs

In this section, let *G*=(*V*,*E*) be an Enredo graph structure and *M*_*G*_=(*G*,*ℓ*) be an Enredo graph model. In an Enredo graph, the set of edges *E*=*E*_*A*_∪*E*_*B*_ decomposes again into a set of directed adjacency edges *E*_*A*_ and a set of undirected block edges *E*_*B*_. We define *ℓ* as a labeling function of the block edges *E*_*B*_.

The block edges *E*_*B*_ of *G* represent blocks, and vertices *V* of *G* represent the ends of blocks. In contrast to alignment graphs, a single block edge represents an entire block. For every block $b\in B_{G}$, there are two vertices in *V* connected by an undirected block edge *e*_*b*_∈*E*_*B*_ (black edges in Figure 4). The function $\ell :E_{B}\to B_{G}$ labels each block edge *e*_*b*_∈*E*_*B*_ with the corresponding block $b\in B_{G}$ such that *ℓ*(*e*_*b*_)=*b*. By choosing one of the two possible block representations as label, the two vertices that are connected by *e*_*b*_ are implicitly labeled as head and tail of the block. Note that they are not labeled as head or tail in *G*. Given the block labels, recovering $B_{G}$ from *M*_*G*_ is again straightforward.

Directed adjacency edges *E*_*A*_ of *G* (colored edges in Figure 4) represent adjacencies. Given any pair of block edges *e*_1_={*u*_*t*_,*u*_*h*_} and *e*_2_={*v*_*t*_,*v*_*h*_} and their labels *ℓ*(*e*_1_)=*b*_1_ and *ℓ*(*e*_2_)=*b*_2_, there is a directed edge *e*∈*E*_*A*_ from an endpoint of *e*_1_ to an endpoint of *e*_2_ for every two adjacent segments *s*_1_=(*p*_1_,*q*_1_) and *s*_2_=(*p*_2_,*q*_2_) with *s*_1_∈*b*_1_ and *s*_2_∈*b*_2_. In contrast to alignment graphs and A-Bruijn graphs, the endpoints of adjacency edges in *G* indicate in relation to other adjacency edges the orientation of segments in a block. Given labels, one endpoint of each block edge is a head vertex and the other a tail vertex. If *p*_1_<*q*_1_, then the adjacency edge *e* starts at the head vertex *u*_*h*_, and if *p*_1_>*q*_1_, *e* starts at the tail vertex *u*_*t*_. If *p*_2_<*q*_2_, then *e* points to the tail vertex *v*_*t*_, and if *p*_2_>*q*_2_, *e* points to the head vertex *v*_*h*_. In other words, *e*=(*u*_*h*_,*v*_*t*_) if *q*_1_=*p*_2_, *e*=(*u*_*h*_,*v*_*h*_) if *q*_1_=*q*_2_, *e*=(*u*_*t*_,*v*_*t*_) if *p*_1_=*p*_2_, and *e*=(*u*_*t*_,*v*_*h*_) if *p*_1_=*q*_2_. Again, there may be several adjacency edges connecting the same two vertices. Thus, the Enredo graph is also a multi-graph.

Due to its two-vertex concept, the structure of an Enredo graph *G* reflects the relative orientation of blocks as opposed to the alignment graph structure and the A-Bruijn graph structure (see Figure 5). *G* is capable of displaying inversions. But just like A-Bruijn graphs, threading a genome with duplications through *G* can be ambiguous (see Figure 6). The path from threading a genome through *G* alternates between block and adjacency edges. Therefore, only multiple occurrences of a block in the same orientation create ambiguity in *G*.

To resolve ambiguity of *G*, we define the sparse labeling function $\ell^{\text{dup}}:E_{A}\to \mathbb{N}$ as a total ordering on the adjacency edges. As for A-Bruijn graphs, we can use the labeling function *ℓ* to determine the adjacency position of an edge *e*∈*E*_*A*_. The function *ℓ*^*d* *u* *p*^ assigns again numbers to the edges *E*_*A*_ such that

$$\ell^{\text{dup}}(e_{1})<\ell^{\text{dup}}(e_{2})\;\text{if}\;a_{1}<a_{2},$$

where *a*_1_ is the adjacency position of *e*_1_, and *a*_2_ is the adjacency positions of *e*_2_. As an example, we use again Figure 6 with labels 1 through 8: One of the edges from the head of A to the tail of B would be labeled with 1; for genome ABDABCEBC, the edge from the head of B to the tail of D would be labeled with 2, and for genome ABCEBDABC, the edge from the head of B to the tail of C would be labeled with 2; and so on.

We generalize the Enredo graph compared to its original definition [39] in some aspects. Enredo graphs originally consider blocks of size 1 as adjacencies: Instead of a block edge with two end vertices that are connected to the rest of the graph by two adjacency edges, the Enredo method only adds a single adjacency edge labeled with a segment. This requires another function *ℓ*_*A*_:*E*_*A*_→*S* that labels adjacency edges *E*_*A*_ with segments *S*. In addition, in the initial phase of the Enredo method segments on adjacency edges between the same two blocks are assumed to be homologous. Because of this assumption and to distinguish non-homologous multi-edges later on, the Enredo method prefers the multi-graph representation with multiplicity labels on one adjacency edge over multiple separate edges. We argue that all segments that are assumed to be homologous should be defined as blocks. Consequently, our description with blocks of size 1 is valid and even simplifies the exposition of the method.

Furthermore, the Enredo method only adds edges for adjacencies that are shorter than a predefined threshold. This results already in a partial segmentation of the genomes bearing several segments per genome in the graph. Parts of the genomes may not be represented. We add all adjacencies to the graph and leave it to later stages to modify the graph.

In the transformations below, we include the replacement of labeled adjacencies by blocks of size 1. The transformation from an Enredo graph structure to an A-Bruijn graph structure is possible without additional labels. The other direction, from A-Bruijn graphs to Enredo graphs, requires additional information about inversions as shown by the example in Figure 5.

###### *Enredo graphs from A-Bruijn graphs*

First, we describe the transformation of an A-Bruijn graph structure *G*^′^=(*V*^′^,*E*^′^) into an Enredo graph structure *G*=(*V*,*E*_*B*_∪*E*_*A*_) using the labeling function *ℓ*^*i* *n* *v*^:*E*^′^→{+,−}×{+,−}. Then, we describe the transformation of blocks of size 1 to labeled adjacency edges in the Enredo graph given full block information by the function *ℓ* for transferring labels.

To transform *G*^′^ into *G*, add for each A-Bruijn graph vertex *v*^′^∈*V*^′^ a tail vertex *v*_*t*_ and a head vertex *v*_*h*_ to the set of Enredo graph vertices *V*. Additionally, add an undirected edge *e*_*b*_ between *v*_*t*_ and *v*_*h*_ to the set of Enredo graph block edges *E*_*B*_. We obtain a one-to-one mapping of A-Bruijn graph vertices and Enredo graph block edges, which we keep as separate labels *m*:*V*^′^→*E*_*B*_ on A-Bruijn graph vertices such that *m*[ *v*^′^]=*e*_*b*_.

Using the labeling function *ℓ*^*i* *n* *v*^ and the mapping *m*, we can unambiguously transfer adjacency edges to the Enredo graph. For each edge *e*^′^=(*u*^′^,*v*^′^) in the set of A-Bruijn graph edges *E*^′^ where *m*[ *u*^′^]=*e*_*u*_ and *m*[ *v*^′^]=*e*_*v*_, add an edge *e*=(*u*_*x*_,*v*_*y*_) to the set of Enredo graph adjacency edges *E*_*A*_ where *u*_*x*_ is an endpoint of *e*_*u*_ and *v*_*y*_ is an endpoint of *e*_*v*_. The vertex *u*_*x*_ is the head vertex of *e*_*u*_ if the first bit in *ℓ*(*e*^′^) is +, and otherwise the tail vertex. The vertex *v*_*y*_ is the tail vertex of *e*_*u*_ if the second bit in *ℓ*(*e*^′^) is +, and otherwise the head vertex.

In another step, we can transform all block edges *e*_*b*_={*v*_*t*_,*v*_*h*_} representing blocks of size 1 into adjacency edges. Since the size of *ℓ*(*e*_*b*_) is 1, the corresponding vertices *v*_*t*_ and *v*_*h*_ are incident to exactly one adjacency edge each, *e*_1_=(*u*_*x*_,*v*_*t*_) and *e*_2_=(*v*_*h*_,*w*_*y*_). Replace such sets of two vertices *v*_*t*_, *v*_*h*_ and three edges *e*_1_, *e*_2_, *e*_*b*_ by a new adjacency edge *e*=(*u*_*x*_,*w*_*y*_). Finally, transfer the label of the block edge *ℓ*(*e*_*b*_)={*s*} to the adjacency edge such that *ℓ*_*A*_(*e*)=*s*.

###### *Enredo graphs to A-Bruijn graphs*

We start by describing how to recover block edges for blocks of size 1 from adjacency edges that are labeled with segments by *ℓ*_*A*_ in an Enredo graph structure *G*=(*V*,*E*_*B*_∪*E*_*A*_). Afterwards, we describe the transformation from *G* to an A-Bruijn graph structure *G*^′^=(*V*^′^,*E*^′^), which is possible without additional labels.

Replace each edge *e*=(*u*_*x*_,*v*_*y*_) from the set of Enredo graph adjacency edges *E*_*A*_, where *ℓ*_*A*_(*e*)=*s*, by two vertices *v*_*t*_ and *v*_*h*_ and a block edge *e*_*b*_=(*v*_*t*_,*v*_*h*_), and set *ℓ*(*e*_*b*_)={*s*}. Further, add *e*_1_=(*u*_*x*_,*v*_*t*_) and *e*_2_=(*v*_*h*_,*v*_*y*_) to the set of Enredo graph adjacency edges *E*_*A*_.

For the transformation to an A-Bruijn graph, add for each edge *e*_*b*_=(*v*_*h*_,*v*_*t*_) in the set of Enredo graph block edges *E*_*B*_, a vertex *v*^′^ to the set of A-Bruijn graph vertices *V*^′^. Again, we obtain a one-to-one mapping of Enredo graph block edges and A-Bruijn graph vertices, which we keep this time as labels *m*:*E*_*B*_→*V*^′^ on Enredo graph block edges such that *m*[ *e*_*b*_]=*v*^′^. Finally, add for each edge *e*=(*u*_*x*_,*v*_*y*_) in the set of Enredo graph adjacency edges *E*_*A*_ where *u*_*x*_ is incident to the block edge *e*_*u*_ and *v*_*y*_ is incident to the block edge *e*_*v*_, an edge *e*^′^=(*u*^′^,*v*^′^) to the set of A-Bruijn graph vertices, where *m*[ *e*_*u*_]=*u*^′^ and *m*[ *e*_*v*_]=*v*^′^. In this last step, we lose inversion information in the graph’s structure.

##### Cactus graphs

In this section, let *G*=(*V*,*E*) be a cactus graph structure and *M*_*G*_=(*G*,*ℓ*) be a cactus graph model. Cactus graphs have only one type of edges. We define *ℓ* as a labeling function of the edges *E*. The cactus graph structure *G* stands out from the other graph structures by fulfilling well-defined structural properties: Every edge *e*∈*E* is part of at most one simple cycle, which makes *G* a cactus graph [41], and *G* has an Eulerian circuit [43]. A number of construction steps guarantee these properties.

Let *A* be the set of all adjacencies of segments. The vertices *V* of *G* partition *A* into a set of pairwisely disjoint subsets *Ω*: Each element *ν*∈*Ω* is a subset of *A*, *μ*∩*ν*=*∅* for any two sets *μ*,*ν*∈*Ω*, and $\underset{\nu \in A}{⋃}\nu =A$. For each subset *ν*∈*Ω*, there is a vertex in *V*. In addition, *G* has one distinct vertex, the *origin*, that represents the start and end of all genomes *ϕ*. In Figure 4, for example, the vertex *ϕ* represents the start and end, the vertex *α*∪*β* represents a subset of twelve adjacencies, the vertex *γ* represents a subset of eleven adjacencies, and the vertex *δ* represents a subset of seven adjacencies. In the vertex *δ*, for example, three of the adjacencies are from the red genome, two from the blue genome, and another two from the green genome as shown in the enlarged vertices. The subsets correspond to subgraphs of the Enredo graph (shaded in gray in Figure 4). All adjacencies at one end of a block are always part of the same subset. We describe the details on how to determine the subsets below in the transformation from Enredo graphs.

The edges *E* of *G* represent blocks just like block edges in Enredo graphs. For each block $b\in B_{G}$, there is an undirected edge *e*={*u*,*v*} in the set of cactus graph edges *E* (black lines in Figure 4). The endpoint *u*∈*V* of *e* represents a subset of adjacencies *μ*∈*Ω* that contains all adjacencies at one end of *b*, and the other endpoint *v*∈*V* represents *ν*∈*Ω* that contains all adjacencies at the other end of *b*. It is possible that *u*=*v*. The function $\ell :E\to B_{G}$ labels each block edge *e*∈*E* with the corresponding block $b\in B_{G}$ such that *ℓ*(*e*)=*b*. With this labeling, recovering $B_{G}$ from *M*_*G*_ is again straightforward.

The cactus graph has no directed edges as found in other graphs. Since vertices of *G* represent segment adjacencies in sets, the size of blocks and the number and precise set of adjacencies remain unclear in the structure. Recovering this information from *G* is impossible as the following examples from Figure 4 demonstrate: The cactus graph structure does not tell how many genomes traverse block F and whether block I and K are adjacent in one of the genomes or not.

Still, each genome corresponds to a (not necessarily simple) path through *G*. With the help of labels we can recover this path. The colored lines in the enlarged vertices in Figure 4 provide the equivalent information as colored adjacency edges in Enredo graphs and would resolve ambiguity for threading if no duplications were present. More information is necessary to resolve all ambiguity. We suggest $\ell^{\text{adj}}:E\to 2^{\{+,-\}\times \mathbb{N}}$, where 2^*X*^ denotes the power set of a set *X*, to label the edges with lists of pairs of an orientation bit and a positive number. The lists have an entry for each segment of the blocks *b*=*ℓ*(*e*). The orientation bits are necessary to determine the relative orientation of segments within blocks that are represented by edges *e*=(*v*,*v*) (see blocks B, C, D, F, G, H, and J in Figure 4). The numbers impose a strict total ordering ≺ on all genome segments *s*_1_,*s*_2_∈*S* where *s*_1_≺*s*_2_ if *s*_1_ is left of *s*_2_.

Cactus graphs are not as independently used as the other genome alignment graphs. The cactus method operates on two graphs, the cactus graph and another graph called the adjacency graph [40]. Interestingly, the latter has the same structure as an Enredo graph. We view the cactus graph, which enables the characterization and detection of new substructures, as a supergraph on top of the Enredo graph. The transformation of Enredo graph structures to cactus graph structures conforms with the construction of a cactus graph [22,40] and does not require additional labels. The transformation back to Enredo graphs is ambiguous as the above mentioned examples from Figure 4 show. For this reason, our description of this transformation uses the sparse labeling *ℓ*^*a* *d* *j*^ in addition to the graph structure.

###### *Cactus graphs from Enredo graphs*

To transform an Enredo graph structure $G^{\prime }=(V^{\prime },E_{B}^{\prime }\cup E_{A}^{\prime })$ into a cactus graph structure *G*=(*V*,*E*), we follow three steps described in [22,40]. First, we transform the Enredo graph into a precursor cactus graph. The second and third steps modify the precursor to ensure the structural properties of cactus graphs. The second step guarantees that every edge is part of at most one simple cycle. After the third step, the graph is Eulerian. Throughout all steps, we make use of a many-to-one mapping *m*:*V*^′^→*V* from Enredo graph vertices *V*^′^ to cactus graph vertices *V*, which labels each Enredo graph vertex *v*^′^∈*V*^′^ with a cactus graph vertex *v*∈*V* such that *m*[ *v*^′^]=*v*.

First, compute all *adjacency-edge connected components*$C_{A}$ in the Enredo graph structure *G*^′^. Each component $C\in C_{A}$ is a subset of the vertices *V*^′^. For each $C\in C_{A}$, add a vertex to the set of cactus graph vertices *V*. Assuming that the start and end of all genomes are connected, add only one origin vertex for all of them to *V*. We obtain the many-to-one mapping that indicates the cactus vertex representing the adjacency edge connected component of any Enredo graph vertex. Given this mapping, transfer the Enredo graph block edges *E*_*B*_ to the cactus graph: For each edge *e*^′^={*u*^′^,*v*^′^} in the set of Enredo graph block edges *E*_*B*_, where *m*[ *u*^′^]=*u* and *m*[ *v*^′^]=*v*, add an edge *e*={*u*,*v*} to the set of cactus graph edges *E*. It is possible that *u*=*v* even if *u*^′^≠*v*^′^. This yields the precursor cactus graph in Figure 4.

In the second step, remove sets of vertices from *V* that are 3-edge-connected and add instead a single vertex *v* to *V* (vertices *α* and *β* in Figure 4). Correct the mapping *m* and redirect block edges that were incident to any vertex in the 3-edge connected component, to be incident to *v*.

Finally, replace connected components formed only by edges whose removal disconnect the graph (not present in Figure 4). Each such component is a tree with leaf and branching vertices *v*_1_,…,*v*_*c*_. Remove *v*_1_,…,*v*_*c*_ and add instead a new vertex *v* to *V*. Just as before, correct the mapping *m* and redirect incident block edges to *v*.

###### *Cactus graphs to Enredo graphs*

In the transformation from a cactus graph structure *G*=(*V*,*E*) to an Enredo graph structure $G^{\prime }=(V^{\prime },E_{B}^{\prime }\cup E_{A}^{\prime })$, we use the labels *ℓ*^*a* *d* *j*^ to separate the sets of adjacencies represented by cactus graph vertices *V* and to add edges $E_{A}^{\prime }$ that represent single adjacencies to the Enredo graph structure. In addition, we create a one-to-one edge mapping $m:E\to E_{B}^{\prime }$ that labels each cactus graph edge *e*∈*E* with an Enredo graph block edge $e_{b}^{\prime }\in E_{B}^{\prime }$ where $e_{b}^{\prime }=\{u^{\prime },v^{\prime }\}$. In this mapping, we store a direction of each Enredo graph block edge and distinguish the tail vertex *u*^′^ from the head vertex *v*^′^ such that *m*[ *e*]=(*u*^′^,*v*^′^). The direction is not present in the Enredo graph structure *G*^′^. The transformation proceeds by threading the genomes  through *G*.

Initially, identify among all cactus graph edges incident to the origin vertex *u*∈*V* the edge *e*_0_={*u*,*v*} whose label contains the smallest number *n*_0_∈*ℓ*^*a* *d* *j*^(*e*_0_) where *n*_0_<*n* and *n*∈*ℓ*^*a* *d* *j*^(*e*) with *e*={*u*,*x*}. Add two vertices *u*^′^ and *v*^′^ to the set of Enredo graph vertices *V*^′^ and an edge $e_{b}^{\prime }=\{u^{\prime },v^{\prime }\}$ to the set of Enredo graph block edges $E_{B}^{\prime }$. Update the mapping such that *m*[ *e*_0_]=(*u*^′^,*v*^′^). Keep a reference to *v*^′^ for the next step.

Among all edges incident to *v*, identify the next edge *e*_1_=(*v*,*w*) whose label contains the next larger number *n*_1_∈*ℓ*^*a* *d* *j*^(*e*_1_) such that *n*_1_>*n*_0_ but *n*_1_<*n* where *n*≠*n*_0_ and *n*∈*ℓ*^*a* *d* *j*^(*e*) with *e*={*v*,*x*}. If the mapping for *e*_1_ is undefined, add two vertices *v*^′′^ and *w*^′^ to the set of Enredo graph vertices *V*^′^ and an edge $e_{b}^{\prime }=\{v^{\prime \prime },w^{\prime }\}$ to the set of Enredo graph block edges $E_{B}^{\prime }$. Update the mapping such that *m*[ *e*_1_]=(*v*^′′^,*w*^′^). Further, add an edge $e_{a}^{\prime }=(v^{\prime },v^{\prime \prime })$ to the set of Enredo graph adjacency edges *E*_*A*_ and keep *w*^′^ for the next step. If the mapping for *e*_1_ is already defined with *m*[ *e*_1_]=(*v*^′′^,*w*^′^), only add an adjacency edge: If the orientation bit in *ℓ*^*a* *d* *j*^(*e*_1_) is +, add the edge $e_{a}^{\prime }=(v^{\prime },v^{\prime \prime })$ to the set of Enredo graph adjacency edges *E*_*A*_ and keep *w*^′^ for the next step. If the orientation bit is −, add an edge $e_{a}^{\prime }=(v^{\prime },w^{\prime })$ and keep *v*^′′^ for the next step.

Next, repeat the same for incident edges of *w*. Proceed like this until reaching the end of all genomes to obtain the full Enredo graph structure *G*^′^.

All in all, the need for labels shows that the four graphs markedly differ in the information represented in their structures. Complete duplication information (dup) is only present in alignment graph structures, and only the structure of Enredo graphs reveals inversion information (inv). A-Bruijn graphs are a compact and intuitive representation but lack both inversion and duplication information. Finally, cactus graph structures do not represent parts of the adjacency information (adj). Despite these structural differences, all graph models, which include labels, can be transformed into each other.

Based on these observations, some advantages or disadvantages of the graph structures become apparent. For example, for a genome aligner intended to reveal inversions, an Enredo graph structure appears to be more suitable, whereas a more general analysis of the genetic content of genomes will work well with the more compact A-Bruijn graph structure. Duplications are best visible in an alignment graph structure. The advantage and information provided by cactus graphs subdivides genomes into independent regions revealing specific and unique substructures as described in the following.

### Graph substructures

We collected substructures from graph-based genome alignment approaches and classify them here into four types: colinear paths, visiting blocks, short cycles, and cactus groups. Substructures are useful for deriving a meaningful genome segmentation or they indicate misalignment, i. e., the alignment of non-homologous segments. Furthermore, they pinpoint parts of genome alignments that can be improved through modification.

Some substructures have been described for several graph-based approaches, while others are unique to only one approach. We conjecture that it is possible to identify all substructures in all graph *models*. If the time complexity for detecting occurrences of the substructures was the same in all graphs models, they could be used interchangeably. Here, our aim is to analyze abilities of the graph *structures* to reveal potential misalignments without additional information from labels.

#### 

##### Colinear paths

We refer to the first type of substructures as *colinear paths*. Colinear paths are sets of blocks that appear in one or more genomes consecutively in the same orientation and without breakpoints in between. A sequence of blocks *b*_1_,…,*b*_*k*_ forms a colinear path if there is an adjacency but no breakpoint between *b*_*i*_ and *b*_*i*+1_ for all *i*=1,…,*k*−1. Consequently, all blocks along a colinear path have the same size and there are segments *s*_1_∈*b*_1_ and *s*_*k*_∈*b*_*k*_ with *s*_1_=(*p*_1_,*q*_1_) and *s*_*k*_=(*p*_*k*_,*q*_*k*_) such that *s*=(*p*_1_,*q*_*k*_) is a consecutive genome segment that concatenates one segment from each block *b*_1_,…,*b*_*k*_. We also consider a single block as a colinear path.

A colinear path is *maximal* if it cannot be further extended by other adjacent blocks, but is bounded by breakpoints. Note that alignment modifications often remove bounding breakpoints such that a colinear path can again be further extended. The set of maximal colinear paths of a genome alignment determines the final genome segmentation. Independent from the underlying graph structure, all graph-based genome alignment methods have the common aim to maximize colinear paths both in terms of size (number of genome segments) and length (total segment lengths).

In Enredo graphs, simple non-branching paths are colinear paths. Similarly, colinear paths appear as non-branching paths in the A-Bruijn graph structure, but here a non-branching path is not necessarily a colinear path. Along a non-branching path in A-Bruijn graphs, one or more blocks can be inverted in a subset of the genomes. The structure of A-Bruijn graphs provides no information about inversions (see Figure 5). Thus, to detect colinear paths in A-Bruijn graphs, information from labels is necessary. Only a single vertex is detectable as (not necessarily maximal) colinear path in the structure of A-Bruijn graphs. The same holds for the alignment graph structure: The detection of consecutive blocks is straightforward, but in order to avoid the inclusion of inverted blocks that break colinearity, additional information about inversions is necessary. And finally, colinear paths appear in the cactus graph structure as non-branching paths although non-branching paths are not necessarily colinear paths.

##### Visiting blocks

We name the second type of substructure *visiting block*, which conceptually is a special type of a maximal colinear path. A maximal colinear path {*b*_1_,…,*b*_*k*_} is a visiting block if there is a block *b*_0_ adjacent to *b*_1_ and a block *b*_*k*+1_ adjacent to *b*_*k*_ with the following two symmetric conditions (without loss of generality, we assume that the tail of *b*_0_ is adjacent to the head of *b*_1_ and the tail of *b*_*k*_ is adjacent to the head of *b*_*k*+1_): For all segments *s*=(*p*_1_,*q*_*k*_) of the colinear path that are adjacent at position *p*_1_ to a segment *s*_0_∈*b*_0_, there is a segment *s*_*k*+1_∈*b*_*k*+1_ adjacent to *s* at position *q*_*k*_; and for all segments *s*=(*p*_1_,*q*_*k*_) that are adjacent at position *q*_*k*_ to a segment *s*_*k*+1_∈*b*_*k*+1_, there is a segment *s*_0_∈*b*_0_ adjacent to *s* at position *p*_1_. The important property is that all segments from block *b*_0_ that are adjacent to segments of the colinear path, continue in the same block *b*_*k*+1_ at the other end of the colinear path and vice versa.

A visiting block arises from merging blocks from within a colinear path with other blocks. If the merged blocks are short, they often only have spurious similarity. Hence, they break colinearity at two positions without providing much evidence for a large structural change. This is a reason why genome aligners address visiting blocks and separate the otherwise colinear paths.

**Figure 8:**
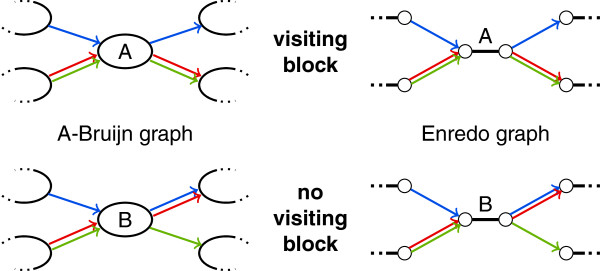
**Visiting blocks are not distinguishable in the structure of A-Bruijn graphs and in the structure of Enredo graphs.** In this example, only the colors of adjacency edges reflect a difference between the substructure at the top (visiting block) and at the bottom (no visiting block). We consider colors as edge labels, which are not present in the graph structures. Thus, visiting blocks do not form unique substructures in the structures of A-Bruijn and Enredo graphs.

In A-Bruijn and Enredo graphs, visiting blocks appear as simple non-branching paths bounded by branching vertices. In Enredo graphs, the path always starts and ends with a block edge. In both graphs, at least one branch which enters the visiting block at one end must be formed by a set of segments that leaves the visiting block as its own separate branch at the other end (see Figure 8). This condition makes it impossible to identify visiting blocks in the A-Bruijn and Enredo graph structures. Likewise, the structure of cactus graphs alone does not reveal visiting blocks. Only in the structure of alignment graphs, it is possible to determine whether a given colinear path is a visiting block or not.

Visiting blocks have been described for A-Bruijn graphs as microblocks [38] and also for Enredo graphs both implicitly in the “joining” operation and explicitly as a first type of “aberrant homologies” [39]. Furthermore, we view another type of “aberrant homologies” from Enredo graphs as a special case of this substructure: retrotransposed pseudogenes that cause a series of successive visiting blocks.

##### Short cycles

Cycles in genome alignments are indicators for rearrangement. A change in one of two identical genomes often introduces a cycle in the corresponding genome alignment. In the same way, spurious similarity causes cycles and breaks colinearity. If there are many cycles, they often hide significant colinearity. For this reason, many genome aligners eliminate short cycles.

Specific types of cycles also play a role for colinear sequence alignment. For example, alignment graphs without mixed cycles are colinear alignments [33]. Thus, we can compute colinear alignments by eliminating mixed cycles from alignment graphs. Similarly, the partial order alignment (POA) program [44] uses directed acyclic graphs (DAGs) for alignment representation, essentially A-Bruijn graphs without directed cycles.

We define a genome alignment cycle as a sequence of blocks *b*_1_,…,*b*_*k*_ where block *b*_*i*_ is adjacent to block *b*_*i*+1_ for all *i*=1,…,*k*−1 and *b*_*k*_ is adjacent to *b*_1_. Further, we require all sets of positions that define adjacencies between two blocks *b*_*i*_ and *b*_*i*+1_ along the cycle to be disjoint. Thereby we exclude pairs of adjacent blocks from the set of genome alignment cycles. A cycle is short if the total length of segments along the cycle is below a given length threshold.

**Figure 9:**
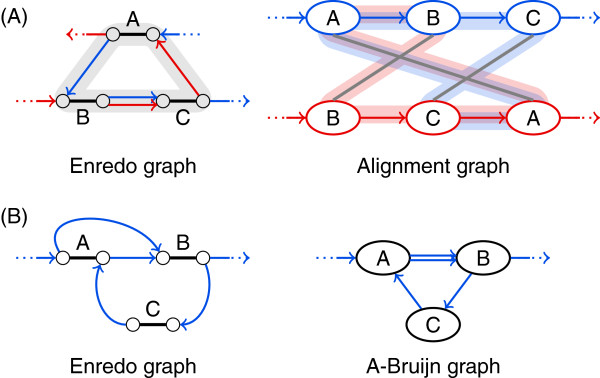
**Genome alignment cycles have a one-to-one correspondence only in the structure of Enredo graphs.(A)** A cycle in the Enredo graph structure may correspond to several overlapping cycles in the alignment graph structure. In this example, two cycles in the alignment graph structure are shaded in red and blue. **(B)** Cycles caused by inversions appear only in the Enredo graph structure. In this example, the upper cycle in the Enredo graph structure is due to an inversion in block A, hence, does not appear in the A-Bruijn graph structure.

The definition of genome alignment cycles corresponds to simple mixed cycles in the Enredo graph structure. They mostly appear in the A-Bruijn graph structure and alignment graph structure as (mixed) simple cycles, too, but there is no one-to-one correspondence: The alignment graph structure can have more than one cycle for a single genome alignment cycle (see Figure 9A); and genome alignment cycles that are caused by inversions are not visible in the alignment graph structure and A-Bruijn graph structure. Figure 9B shows an example for two genome alignment cycles that appear as a single cycle in the A-Bruijn graph structure. Despite these essential differences, cycles in the alignment graph structure, A-Bruijn graph structure, and Enredo graph structure always correspond to genome alignment cycles as opposed to cycles in the cactus graph structure. Subgraphs in the structures of alignment graphs, A-Bruijn graphs, and Enredo graphs that correspond to cycles in the cactus graph structure are not even necessarily connected (see below).

In the following, we discuss two characteristics for discriminating between different types of cycles, the orientation of adjacencies and the number of maximal colinear paths. Next, we briefly address the special case of palindromes. In addition, we describe how simple cycles in cactus graphs are used as characteristic substructures although they differ from genome alignment cycles.

###### *Orientation of adjacencies*

A-Bruijn graphs represent adjacencies as directed edges. This allows classifying cycles into those that follow the direction of edges and those that ignore the direction of edges. Pevzner and colleagues refer to the two types of cycles as whirls and bulges [19,35]. Whirls are directed, and bulges ignore the direction of edges. The graph-based genome aligner ABA addresses whirls and bulges in A-Bruijn graphs [19].

**Figure 10:**
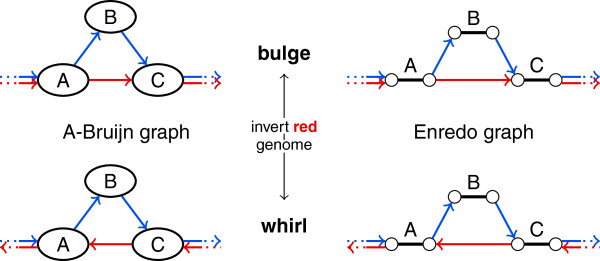
**The classification of cycles into whirls and bulges depends on the orientation of genomes.** In this example, inversion of the red genome transforms the cycle from bulge to whirl or vice versa.

The classification of cycles in whirls and bulges becomes ambiguous when the graph represents multiple genomes. It depends on the initially chosen relative orientations of the genomes. If we invert a subset of the genomes, some whirls become bulges and some bulges become whirls (see Figure 10). Note that whirls and bulges have been first introduced for repeat resolution within *one* genome [35], where the classification in whirls and bulges is unambiguous.

###### *Number of maximal colinear paths*

A genome alignment cycle is formed by complete maximal colinear paths and possibly single additional blocks. For example, the cycle in Figure 10 is formed by the maximal colinear path consisting of the single block *B* and two additional blocks *A* and *C*. *A* and *C* may be part of longer maximal colinear paths. In contrast to the orientation of adjacencies, the number of maximal colinear paths classifies the cycles unambiguously [38].

The A-Bruijn graph based approach DRIMM-Synteny [38] uses a classification of cycles into one-way, two-way, and composite cycles, which is similar but not equivalent to a classification according to the number of maximal colinear paths. DRIMM-Synteny focuses only on one-way and two-way cycles even though there can be cycles formed by more than two paths. The “annealing” operation in Enredo [39] places special emphasis on cycles formed by two maximal colinear paths after each of these paths has been joined to a single adjacency edge. In addition, Enredo addresses all other cycles as the third type of “aberrant homologies”.

###### *Palindromes*

**Figure 11:**
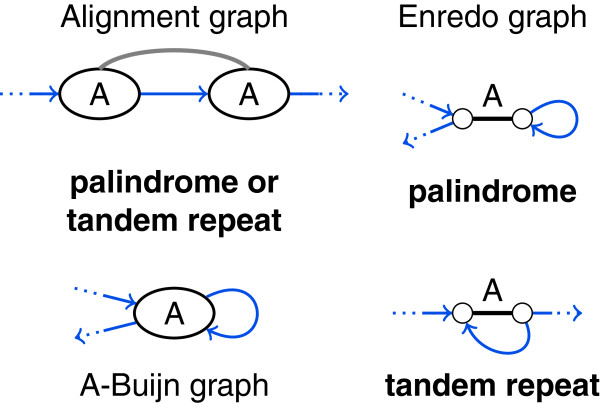
**Palindromes and tandem repeats are not distinguishable in the structure of alignment and A-Bruijn graphs.** Depending on the relative orientation of the segments in block A, the displayed alignment graph structure and A-Bruijn graph structure represents a palindrome or a tandem repeat. In Enredo graphs, palindromes and tandem repeats form distinct substructures.

Palindromes in genomes are inverted tandem duplications. Hence, they traverse a duplicated block twice and in both directions. Palindromes create a special type of cycles in genome alignments formed by only one adjacency at one end of a block. For the detection of palindromes and distinction against tandem repeats, inversion information is necessary. Thus, the structure of alignment graphs and A-Bruijn graphs alone cannot reveal palindromes. In Enredo graphs, we recognize palindromes by an adjacency edge loop (see Figure 11). Palindromes are separately addressed as “thorns” in A-Bruin graphs [38] and mentioned as “aberrant homologies” in Enredo [39].

###### *Cactus chains*

In cactus graphs, simple cycles are named *chains*[22]. The corresponding subgraphs of cactus chains in Enredo graphs, A-Bruijn graphs, and alignment graphs are not necessarily connected (see blocks *A*, *E*, *I*, *K* in Figure 4). But even though chains do not correspond to continuous segments of genomes, they represent conserved orders of blocks (e. g., blocks *A*, *E*, *I*, *K* in Figure 4 appear in this order in all genomes). Cactus chains are addressed by the Cactus method.

It is possible to identify the subset of blocks forming a cactus chain in the Enredo graph structure, for the simple reason that an Enredo graph can be transformed into a cactus graph. However, it appears impossible to characterize chains in Enredo graphs without computing e. g., 3-edge connected components. In the structure of alignment and A-Bruijn graphs, information about the orientation of adjacent blocks is missing for identifying cactus chains.

##### Cactus groups

Paten et al. refer to adjacency edge connected components, which are computed for constructing a cactus graph, as *groups*[22]. A cactus group is a set of adjacencies that forms an adjacency-edge connected component in the Enredo graph. All adjacencies of one group are represented by one vertex in the cactus graph structure, but a cactus graph vertex can represent several groups. Strictly speaking, cactus groups are visible in the structure of Enredo graphs but not in the structure of cactus graphs. Similarly, it is not possible to recognize groups in the alignment graph and A-Bruijn graph structures because this requires information about the orientation of adjacent blocks.

In summary, inversion and duplication information is necessary for the complete detection of all substructures. Visiting blocks require duplication information, and all other substructures require inversion information. Hence, none of the four graphs reveals all substructures solely by its structure.

This concludes our classification of substructures on the basis of a not necessarily exhaustive list of substructures. Identification of further substructures or an assessment of their relevance for the accuracy of genome alignments may possibly point towards another way of classifying them.

### Modifications

Graph-based genome aligners modify the genome alignments by eliminating substructures from the graphs. The aim is to reveal long conserved homologies, i. e., blocks of large size and length. As mentioned in the introduction, genome alignment comprises selection of local alignments and segmentation. Here, we describe modifications that eliminate substructures either by modifying the set of local alignments represented in blocks (“splitting blocks” and “merging parallel blocks”) or by determining breakpoint positions that will be part of the final segmentation (“merging consecutive blocks” and “cutting adjacencies”).

These four modifications derive from the mentioned graph-based genome alignment approaches, but they match the operations described for the approaches only in part. Some genome alignment approaches clearly separate block modification and segmentation, other approaches do both tasks together. Similarly, some approaches apply compound operations consisting of several of the modifications described here. Our intention is to provide small modification entities from which it is possible to assemble more complex operations.

We describe every modification on the set of blocks (not on the level of alignment components but on the level of segments). Furthermore, we mention effects of the modifications in the graph structures although they can be applied to a genome alignment independently from a graph structure. We explain how these modifications correspond to operations in the graph-based genome alignment approaches, especially if the correspondence is not obvious. For example, this is the case for DRIMM-Synteny [38], which solves the sequence modification problem (SMP) on A-Bruijn graphs. The method modifies the sequences and determines the segmentation on the modified sequences before transforming the sequences back. We transfer the effects directly to the original sequences and set of blocks, and refer to the modifications accordingly. The four modifications cover all operations described in the programs ABA, DRIMM-Synteny, Enredo, and Cactus.

#### 

##### Splitting blocks

The most prevalent modification is splitting a block by dividing its set of segments into subsets that form new smaller blocks. Formally, the modification replaces a block *b*={*s*_1_,…,*s*_*n*_}, where *n*≥2 is the size of *b*, by two blocks *b*_1_={*s*_1_,…,*s*_*k*_} and *b*_2_={*s*_*k*+1_,…,*s*_*n*_}, 1≤*k*<*n*. The new blocks may have size 1, thus may consist of a single segment. Transferred to the original set of local alignments from which the blocks were formed, this modification corresponds to removing local alignments. In some cases, it is enough to remove a single pairwise local alignment to split a block into two blocks. In other cases, a particular subset of the local alignments needs to be removed simultaneously.

**Figure 12:**
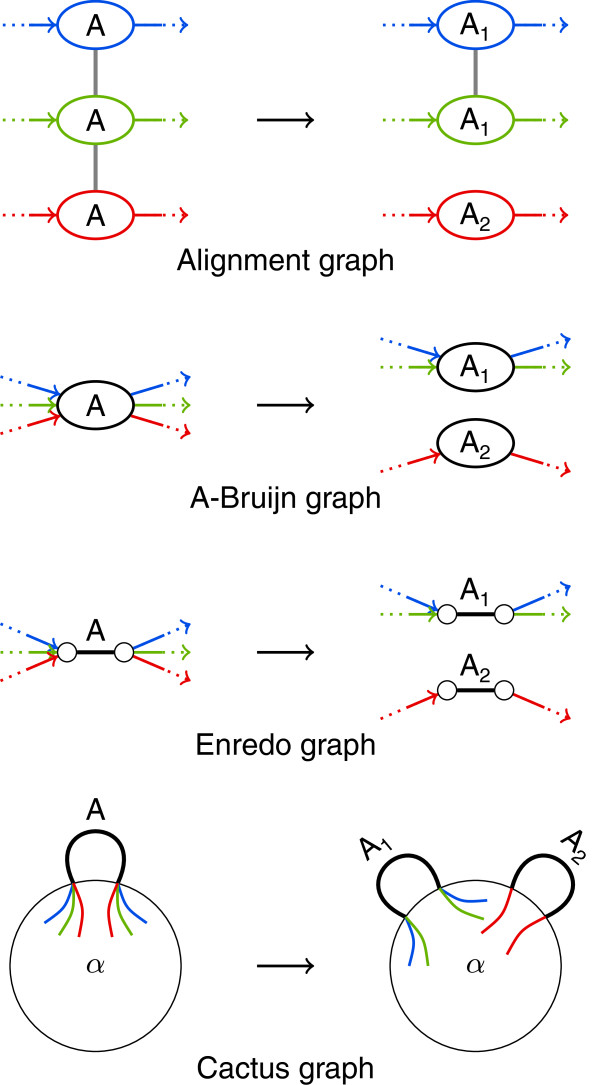
**The effect of splitting a block on the graphs.** In alignment graphs, removal of block edges splits a block if the removal disconnects a block edge connected component. In A-Bruijn graphs and Enredo graphs, vertices (and block edges) need to be multiplied. In cactus graphs, the effect depends on the context. We show only the simplest possibility, where an edge is multiplied.

Splitting blocks has different effects on the genome alignment graphs (see also Figure 12). In the alignment graph structure, the splitting corresponds to removing all edges between two vertex subsets of a block edge connected component. In A-Bruijn graph structures, where vertices represent blocks, the modification replaces a vertex by two new vertices; incoming and outgoing edges are connected to the respective new vertex. The effect of splitting blocks in Enredo graph structures is very similar: The modification duplicates a pair of head vertex and tail vertex connected by a block edge, and reconnects incoming and outgoing adjacency edges accordingly. In cactus graph structures, the splitting of block edges can lead to complex rearrangements with both splitting and merging of vertices.

Graph-based genome aligners eliminate many substructures using this modification. By splitting blocks, we can clearly modify a graph so as to eliminate visiting blocks (see Figure 8), which is done in the programs DRIMM-Synteny and Enredo. While Enredo splits a block into two blocks of arbitrary size, DRIMM-Synteny splits single segments from a block, thus creating blocks of size 1. Additionally, this modification can eliminate small cycles, e. g., whirls in A-Bruijn graphs [35] and mixed cycles in alignment graphs. Further, the sequence modifications in DRIMM-Synteny for one-path cycles and palindromes result in the splitting of segments from blocks. Finally, the “melting” operation in the Cactus method splits all blocks along a cactus chain into blocks of size 1.

##### Merging parallel blocks

The opposite to block splitting is a modification that merges blocks by adding local alignments between segments of the blocks. To merge two blocks *b*_1_={*s*_1_,…,*s*_*k*_} and *b*_2_={*s*_*k*+1_,…,*s*_*n*_} of size *k* and size *n*−*k*, respectively, into a new block *b*={*s*_1_,…,*s*_*n*_} of size *n*, it is sufficient to add a local alignment of two segments *s*_*i*_∈*b*_1_ and *s*_*j*_∈*b*_2_. Such local alignments can be new or previously removed by splitting blocks. Note that merging of parallel blocks implicitly aligns all segments of the two blocks.

The effect on the graph structures is the reverse of block splitting. In the alignment graph structure, it corresponds to adding block edges. In the A-Bruijn graph structure two vertices are replaced by a single vertex. In the Enredo graph structure, two block edges with head and tail vertices are being replaced by a single block edge with one head and one tail vertex. In the cactus graph structure, merging of parallel blocks can lead to complex rearrangements just as splitting of blocks. The result is typically a longer chain or a new sub-cactus.

Graph-based genome alignment approaches usually merge blocks based on the structure of surrounding blocks. Two-way cycles and bulges in A-Bruijn graphs and Enredo graphs are substructures that suggest to merge parallel blocks [38,39]. Furthermore, the genome segments within cactus groups are more likely to be homologous than others, hence, subject to merging [22]. Both in Enredo and in cactus graphs, the modification is termed “annealing”.

##### Merging consecutive blocks

The preceding two modifications often generate new or longer colinear paths. It is possible to replace the consecutive blocks of a colinear path by a new longer block that rules out the possibility of a breakpoint between the merged blocks. The modification replaces two adjacent blocks *b*_1_={*s*_1_,…,*s*_*n*_} and $b_{2}=\{s_{1}^{\prime },\ldots ,s_{n}^{\prime }\}$ without breakpoint in between by a longer block *b* that is formed by the concatenation of all adjacent segments *s*_*i*_ and $s_{i}^{\prime }$ where *i*=1,…,*n*. Merging consecutive blocks does not directly affect the alignment of the genomes, but simplifies the graph structures and also genome segmentation.

The effects on the graphs are straightforward. In the alignment graph structure, a single vertex replaces each pair of vertices in two adjacent block edge connected components. In the A-Bruijn graph structure, one vertex replaces two consecutive vertices. In the Enredo graph structure, one block edge replaces a path consisting of a block edge, adjacency edge, and another block edge. And similarly in the cactus graph structure, one block edge replaces a path of a block edge, a vertex, and another block edge, thereby reducing the number of vertices in a chain.

Merging consecutive blocks is part of the “joining” operation in the Enredo method [39]. The other approaches do not apply this modification.

##### Cutting adjacencies

As opposed to merging consecutive blocks, the last modification fixes a breakpoint in the genome alignment by cutting genomes into several segments. For example, given a block *b*={*s*_1_,…*s*_*n*_} with *s*_*i*_=(*p*_*i*_,*q*_*i*_) where *i*=1,…,*n* and with a breakpoint at the tail of *b*, the modification cuts the genomes at all positions *q*_*i*_. The modification does not affect the set of blocks but rather the set of genomes. Thus, it is part of the genome segmentation process.

Cutting adjacencies corresponds to removing a single edge from an A-Bruijn graph structure, a single adjacency edge from an Enredo graph structure, or a set of adjacency edges from an alignment graph structure. Again, there are multiple possible effects in a cactus graph structure. In the simplest case, the cactus graph structure remains unchanged. In all graphs, the removal of edges can disconnect the graph structures, generating several components that correspond to disjoint sets of genome segments. Thus, it can become impossible to thread the genomes through the graphs without additional effort [35,38].

Cutting adjacencies is used in various ways by genome alignment approaches. The ABA method cuts adjacencies for eliminating bulges from A-Bruijn graphs and the Enredo method for eliminating small cycles in general. In addition, the segmentation processes in A-Bruijn and Enredo graphs implicitly use this modification: In DRIMM-Synteny, segmentation is realized by coloring the graph. In Enredo, it is realized by excluding adjacencies shorter than a given length threshold. Genome segmentation in alignment graphs and cactus graphs has not been described explicitly.

## Discussion and conclusions

We compared four graph data structures and their usage for genome alignment. Our comparison identified that essential pieces of information about duplication and inversion are only present in the structures of some graphs. In addition, we examined substructures in the graph structures that are subject to elimination in various genome alignment approaches, and determined four classes of substructures. We found that information about duplications or information about inversions or even both are necessary for distinguishing any type of substructure in the graphs. Thus, it is indeed essential to keep additional information in labels of the vertices or edges, though the different graphs depend on the labels to a lesser or greater extent. Finally, we reduced the set of operations applied for eliminating substructures from the graphs to four elementary modifications. Overall, it became apparent that many ideas are shared by all graph-based approaches.

**Figure 13:**
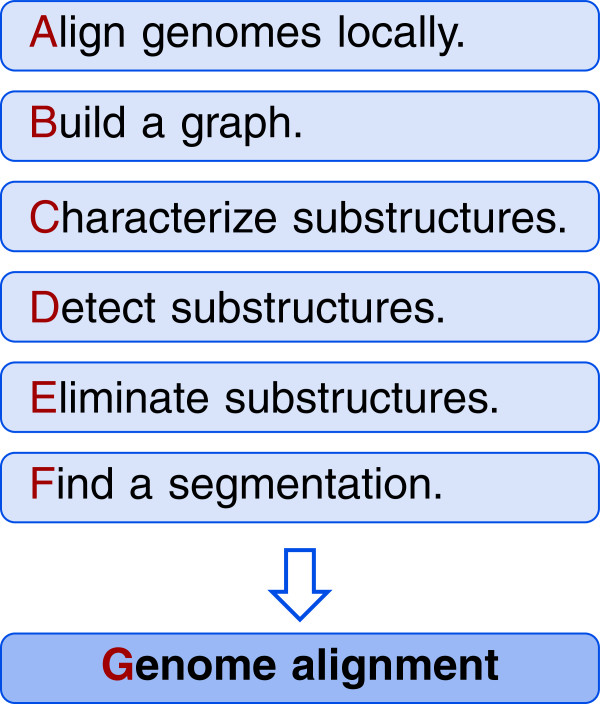
**An ABC to graph-based genome alignment.** These six steps lead to the genome alignment in all of the compared graph-based genome alignment approaches.

These shared ideas allow us to derive a framework for graph-based genome alignment (see also Figure 13), an ABC to G-enome alignment. It begins with the computation of local colinear alignments among the input genomes (A). The choice of the local alignment method is mostly independent from the following steps though it influences the resulting genome alignments. Combining local alignments to blocks, we can build a graph (B). Which graph to choose depends on the respective importance of different substructures for an application. Next, a graph-based genome alignment approach always characterizes a set of graph substructures (C). Substructures sometimes have equivalences in other graphs, but may as well be distinguishable in the structure of only one graph. Detection of all substructure occurrences (D) is a requirement for their subsequent elimination (E). Elimination is accomplished by modifying the underlying set of blocks and sometimes also by introducing breakpoints in the genomes. The breakpoints determine already parts of a genome segmentation, which is finished in a last step (F). The segmentation together with the modified set of blocks defines the genome alignment (G).

This framework describes the main procedure of graph-based genome alignment. Still, it has limitations and there are additional problems to be solved. One such problem addresses blocks and occurs before building a graph. If we do not break up the local colinear alignments into alignment components, blocks may in general partially overlap. It is possible to resolve overlapping blocks by trimming [45] or avoid overlaps by requiring local alignments to be sparse [39]. A good alternative, which is for example used by the genome aligner Mugsy [21], is to obtain a set of mutually disjoint blocks by refining segment matches [46]. A segment match refinement resolves overlaps through modest computation without losing any alignment information.

In addition, the generation of blocks (multiple alignments) from pairwise alignments may pose a problem. There are only few exceptions of genome aligners that avoid the problem by directly computing local multiple alignments [45,47]. If we assume transitivity of the alignment relation, it is straightforward to go from local pairwise alignments to alignment components or to multiple ungapped alignments. In the case of gapped alignments however, pairwise alignments can have conflicting gap patterns. This complicates the task of combining them to a single block. Heuristic methods such as progressive alignment [7] or transitive alignment [9] carry out this task, but are time consuming. Having said that, a colinear realignment for each block carried out after finishing segmentation has proven to significantly improve alignment accuracy [45,48]. This suggests the alternative to ignore gaps in blocks while improving the genome alignment on the level of blocks.

Further, we have not covered all aspects of the framework in this paper and left out details on the detection of substructures. For example, ABA and DRIMM-Synteny detect small cycles by efficiently computing a maximum spanning tree before heuristically inspecting the remaining edges that create cycles. Different detection methods clearly have an influence on the time complexity of an approach and, depending on their sensitivity, also on the accuracy of a genome aligner. Thus, a thorough analysis of detection methods is certainly interesting but beyond the scope of this work.

Similarly, we have not addressed algorithms for eliminating substructures. These algorithms determine the order in which modifications are applied. The elimination of one type of substructures can create other substructures, which again can create the first type of substructures upon elimination. For this reason, iterative elimination strategies are prevalent in graph-based genome aligners. End criteria for iteration are typically given as parameters of the method, e. g., a maximal length of cycles or an explicit number of iterations.

The parameters usually require customized values for every new input set of genomes. Usually, this inhibits broad usage of tools if automatic parameter selection is not offered. A genome aligner has to find a trade-off between size and length of blocks. Very similar genomes will have long blocks conserved across many genomes, whereas more diverged genomes show fewer long blocks and conservation across fewer genomes. Hence, a factor to consider for parameter selection is genome divergence in addition to genome lengths. Given the initial set of local alignments, automatic parameter selection seems possible. It will be necessary to carefully study the influence of all factors to be able to automate the selection, but consequently it will enable a larger community to benefit from graph-based genome aligners.

Finally, graph-based genome aligners, just as other genome aligners, have to decide between positional homology alignment [49] or alignment of all repeats. More precisely, they have to decide, for segments with multiple copies in several genomes, whether to align them in one or in multiple blocks. Not only do repeats lead to a quadratic explosion in the number of pairwise alignments, but they also hide larger regions of colinearity. For this reason, several genome aligners aim at aligning less and predict positional homology [17,21,45]. Graph-based genome aligners compute positional homology to a certain degree. They do not forbid duplications, but separate blocks into positional homologs when splitting blocks.

In conclusion, our framework demonstrates shared aspects of graph-based genome aligners. It contributes to developing a common view on graph-based genome alignment, an active field of research with currently at least two graph-based tools for genome alignment being actively developed [50,51]. In the future, we might identify the steps that have the greatest influence on alignment accuracy. Already now, we believe that the framework provides assistance for the development of new and improved genome aligners.

## Competing interests

The authors declare that they have no competing interests.

## Authors’ contributions

BK and KT participated in the design of the study and drafted the manuscript. MH participated in the design of the study and helped editing the manuscript. KR conceived of the study and participated in its design. All authors read and approved the final manuscript.
